# Self-regulation versus social influence for promoting cooperation on networks

**DOI:** 10.1038/s41598-020-61634-7

**Published:** 2020-03-16

**Authors:** Dario Madeo, Chiara Mocenni

**Affiliations:** Department of Information Engineering and Mathematics, University of Siena, Via Roma 56, 53100 Siena, Italy

**Keywords:** Social evolution, Applied mathematics

## Abstract

Cooperation is a relevant and controversial phenomenon in human societies. Indeed, although it is widely recognized essential for tackling social dilemmas, finding suitable policies for promoting cooperation can be arduous and expensive. More often, it is driven by pre-established schemas based on norms and punishments. To overcome this paradigm, we highlight the interplay between the influence of social interactions on networks and spontaneous self-regulating mechanisms on individuals behavior. We show that the presence of these mechanisms in a prisoner’s dilemma game, may oppose the willingness of individuals to defect, thus allowing them to behave cooperatively, while interacting with others and taking conflicting decisions over time. These results are obtained by extending the Evolutionary Game Equations over Networks to account for self-regulating mechanisms. Specifically, we prove that players may partially or fully cooperate whether self-regulating mechanisms are sufficiently stronger than social pressure. The proposed model can explain unconditional cooperation (strong self-regulation) and unconditional defection (weak self-regulation). For intermediate self-regulation values, more complex behaviors are observed, such as mutual defection, recruiting (cooperate if others cooperate), exploitation of cooperators (defect if others cooperate) and altruism (cooperate if others defect). These phenomena result from dynamical transitions among different game structures, according to changes of system parameters and cooperation of neighboring players. Interestingly, we show that the topology of the network of connections among players is crucial when self-regulation, and the associated costs, are reasonably low. In particular, a population organized on a random network with a Scale-Free distribution of connections is more cooperative than on a network with an Erdös-Rényi distribution, and, in turn, with a regular one. These results highlight that social diversity, encoded within heterogeneous networks, is more effective for promoting cooperation.

## Introduction

Cooperation in human populations is a fundamental phenomenon, which has fascinated many scientists working in different fields, such as biology, sociology, economics^1–6^, and engineering^7–9^. In biology it has been pointed out that the emergence of cooperation may be favored by the presence of kin selection, based on the altruistic behavior among relatives^10,11^. Additionally, many theoretical approaches to understand the evolution of cooperation among non-relatives are based on direct reciprocity; in this case it is assumed that individuals can adopt complex strategies that take into account the past history of their interactions with other individuals^12,13^. Although the previous ones are powerful mechanisms for the evolution of cooperation, they don’t cover peculiar aspects of human behavior. Indeed, the evolution of cooperation leads to reputation building, morality judgement and complex social interactions with ever increasing cognitive demands^14^. These mechanisms are known as indirect reciprocity^15,16^. Other approaches for explaining the emergence of cooperation are based on the presence of norms in a society^17,18^, punishment^19–24^, synergy and discounting^25^, social diversity^26^ and positive interactions^27^. Also imitative processes, based on conformity, have been found to be effective in the promotion of cooperation within a population^28^.

All the aforementioned approaches are based on mathematical models which assume that interactions among players, as well as the update rules of strategies, e.g. when switching between cooperation and defection, are stochastic, and the time evolution of players behavior is described by random variables, e.g. birth-death mechanisms^12^ or payoff optimization through imitation^29^. Specifically, in these models individuals are constrained to choose between cooperation ($C$) and defection ($D$) in a prisoner’s dilemma scenario. Accordingly, the replicator equation is a widely used mathematical model, which represents a deterministic setting ruling the time evolution of the probability distribution of strategies over a well-mixed infinite population^30,31^. However, when dealing with real systems, cooperation may gradually evolve starting from discrete ($C$ or $D$) or fuzzy (intermediate values between $C$ and $D$) initial conditions. In this regard, the *continuous prisoner’s dilemma* has been proposed as a representative model able to account for different levels of cooperation^3,32,33^.

Moreover, the presence of graphs of connections among players is typical for real populations, where asymmetric relationships are frequently observed. The evolution of strategies on graphs has been investigated in several studies^34–42^. Among them, the replicator equation on regular infinite graphs, embedding network reciprocity, has been developed and analyzed in^15,43,44^. Interestingly, the population structure can be assumed to be the result of reputation-driven mechanisms, thus inducing feedbacks favoring the cooperation among individuals^45^.

Recently, the equation for evolutionary game on networks (EGN) has been introduced^46,47^ for modeling the deterministic dynamics of a finite networked population of individuals able to choose strategies in a continuous set. In this framework, people continuously interact over time with all their neighbors, and thus they are able to learn from their environment, composed by friends, colleagues, relatives, and so on. The tools introduced in these papers allow to analyze the dynamics of any single player, and to study more heterogeneous situations, where the initial configuration of the individuals includes partial cooperation. At the same time, the presence of a network of connections among the members of the population introduces some constraints influencing their interactions with neighbors.

In this paper, in order to study the global or partial emergence of cooperation in human societies, we propose to extend the EGN equation described above by introducing self-regulating processes. In cell communication, for example, self-regulation refers to several control mechanisms, such as signal pathways^48^ and quorum sensing^49^, aimed to maintain the healthy state of living systems. Specifically, inspired by^9,50^, where the importance of internal mechanisms in animal societies is discussed, an additional term is added to the EGN equation, accounting for the presence of feedbacks, acting at the level of any single individual. This integration is in agreement with the fact that “humans seem to have an innate tendency to cooperate with one another even when it goes against their rational self-interest^51^”. This characteristic is also recognized as a key factor for explaining the human response to monetary rewards or punishments, where the self-interest is not the only mechanism for decision making^52^.

However, self-regulating mechanisms, encoded by this innate tendency to cooperate, may be in conflict with social pressure. In fact, the presence of conflicts in human decision processes is widely recognized, as reported by Bear and Rand^53^: “In many situations, intuitive and deliberative processes can favor different decisions, leading to inner conflict: Rather than being of a single mind, people are torn between competing desires”.

The extended EGN allows us to study theoretically the emergence of cooperation as the result of the conflict between spontaneous internal factors and social pressure perceived by the members of a social interconnected system. The influence of the network topology is also investigated by extensive simulations.

## The model

Consider a social interconnected system defined by a finite population of players $v=\{1,\ldots ,N\}$ connected through an undirected graph with adjacency matrix ${\bf{A}}=\{{a}_{v,w}\}$. ${\bf{A}}$ is a symmetric $N\times N$ matrix where ${a}_{v,w}=1$ if player $v$ is connected to player $w$, and 0 otherwise. The degree ${k}_{v}={\sum }_{w=1}^{N}{a}_{v,w}$ of player $v$ corresponds to the size of his neighborhood. At each time instant, an individual $v$ will play ${k}_{v}$ continuous prisoner’s dilemma games with his neighbors, thus choosing his own level of cooperation, indicated by ${x}_{v}\in [0,1]$. Pure strategies $C$ and $D$ correspond to ${x}_{v}=1$ and ${x}_{v}=0$, respectively. The replicator dynamics describing this mechanism for two strategies is incorporated by the EGN equation^46,47^, which can be expressed as:

1 $${\dot{x}}_{v}={x}_{v}(1-{x}_{v})\frac{\partial {\phi }_{v}}{\partial {x}_{v}},$$

where the function ${\phi }_{v}$ represents the payoff of player $v$ over the network, thus accounting for the sum of all outcomes of the ${k}_{v}$ two-players games played by $v$ with neighbors (refer to the SI document for further details):

2 $${\phi }_{v}({\bf{x}})=\mathop{\sum }\limits_{w=1}^{N}{a}_{v,w}\phi ({x}_{v},{x}_{w}),$$

where the vector ${\bf{x}}={[{x}_{1},{x}_{2},\ldots ,{x}_{N}]}^{\top }$ represents the strategy profile of the whole population, while $\phi ({x}_{v},{x}_{w})$ is the payoff earned by player $v$ against $w$ when they use strategies ${x}_{v}$ and ${x}_{w}$, respectively. In the specific case of the continuous prisoner’s dilemma game^33^, the payoff function $\phi$ is the following:

$$\phi ({x}_{v},{x}_{w})=(R-T+P-S){x}_{v}{x}_{w}+(S-P){x}_{v}+(T-P){x}_{w}+P,$$

where $R$ is the reward for mutual cooperation, $T$ is the temptation to defect when the opponent cooperates, $S$ is the sucker’s payoff earned by a cooperative player when the opponent is a free rider, and $P$ is the punishment for mutual defection. The social dilemma arises when the temptation to defect is stronger than the reward for cooperation ($T > R$), and the punishment for defection is preferred to the sucker’s payoff ($P > S$). Moreover, $P$ is lower than $R$. Without loss of generality, we assume $R=1$ and $P=0$, thereby normalizing the advantage of mutual cooperation over mutual defection^29^. Under these assumptions, $T > 1$ and $S < 0$, and the payoff function $\phi$ reads as follows:

3 $$\phi ({x}_{v},{x}_{w})=(1-T-S){x}_{v}{x}_{w}+S{x}_{v}+T{x}_{w}.$$

Therefore, the derivative of ${\phi }_{v}$ with respect to ${x}_{v}$, introduced in (1), is:

4 $$\frac{\partial {\phi }_{v}}{\partial {x}_{v}}=\mathop{\sum }\limits_{w=1}^{N}{a}_{v,w}\frac{\partial \phi ({x}_{v},{x}_{w})}{\partial {x}_{v}}=\mathop{\sum }\limits_{w=1}^{N}{a}_{v,w}[(1-T-S){x}_{w}+S].$$

Unlike the standard replicator equation, which deals with the distribution of strategies over a well mixed population of players, indistinguishable from one another except for the strategy chosen, the EGN equation (1) is a system of ODEs, each one describing the strategy evolution of a single player $v$, able to appraise whether a change of his strategy ${x}_{v}$ produces a variation of his own payoff ${\phi }_{v}$. Notice that, since in (1) ${x}_{v}(1-{x}_{v})\ge 0$, the sign of ${\dot{x}}_{v}$ depends only on the term $\partial {\phi }_{v}/\partial {x}_{v}$, which involves the states ${x}_{w}$ of all neighbors, rather than the current state ${x}_{v}$ of player $v$ himself. Then, if this term is positive (negative), player $v$ would like to increase (decrease) his level of cooperation ${x}_{v}$. Of course, when it is null, player $v$ has no incentives to change his mind. In the strict formulation of the prisoner’s dilemma game, unilateral defection is preferred to mutual cooperation, i.e. $T > 1$, and mutual defection overcomes unilateral cooperation, i.e. $S < 0$^29,30,54^. Consequently, in (1), $\partial {\phi }_{v}/\partial {x}_{v}\le 0$, then ${\dot{x}}_{v}\le 0$, showing that the level of cooperation eventually decreases over time towards full defection.

The willingness to pursue cooperation as a greater good may follow from internal mechanisms correlated to personal awareness and culture^51^, contrasting with the aforementioned selfish processes, which ultimately lead to defection. Reasonably, these mechanisms depend on the current strategy ${x}_{v}$ of player himself, and act as inertial terms, which reduce the rational temptation to defect. Inspired by the behavior observed in animal societies^9,50^, we consider an internal mechanism, named self-regulation, defined by the term $-{\beta }_{v}\,{f}_{v}({x}_{v})$, where ${f}_{v}({x}_{v})$ accounts for self-regulation itself, and ${\beta }_{v}$ measures its strength. Self-regulation is meant to balance the external mechanisms $\partial {\phi }_{v}/\partial {x}_{v}$, which in turn indicate the effects of social influence. Notice that, for ${\beta }_{v}=0$, the standard EGN equation is recovered.

**Figure 1:**
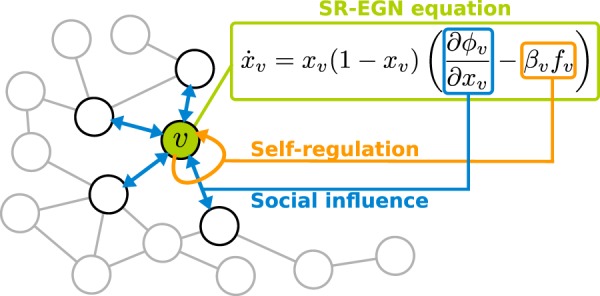
SR-EGN equation. Strategy dynamics of player $v$ (green node) is ruled by the SR-EGN equation (green box). It includes two terms: the social influence term $\partial {\phi }_{v}/\partial {x}_{v}$ (blue box) and the self-regulation term ${\beta }_{v}\,{f}_{v}$ (orange box). The arrows represent the interactions of player $v$ with neighbors (blue) and with himself (orange).

The extended Self-Regulated EGN equation, hereafter called SR-EGN, is reported in Fig. 1. A natural way for defining the function ${f}_{v}$ is to model the self-regulation term as a virtual game that each individual plays against himself, a self-game. For this reason, the game can be a Prisoner’s dilemma game, characterized by the same parameters $T$ and $S$:

5 $${f}_{v}({x}_{v})=(1-T-S){x}_{v}+S.$$

In the SR-EGN equation, positive values of ${\beta }_{v}$ cause an “aware resistance” against the temptation to defect, thus activating a conflict between internal and external mechanisms. Negative values of ${\beta }_{v}$ are not considered since they foster the defective prisoner’s dilemma dynamics. The interplay between social influence and self-regulation constitutes an intuitive explanation of the theoretical results formally proved in the SI document and hereafter schematically presented.

## Results

It is worthwhile to notice that SR-EGN equation can be rewritten as follows:

6 $${\dot{x}}_{v}={x}_{v}(1-{x}_{v})\{{k}_{v}[(1-T-S){\bar{x}}_{v}+S]-{\beta }_{v}[(1-T-S){x}_{v}+S]\},$$

where ${\bar{x}}_{v}=(1/{k}_{v}){\sum }_{w=1}^{N}{a}_{v,w}{x}_{w}$ represents the *equivalent player*, which incorporates the average decisions of the neighbors of player $v$. Since the SR-EGN depends on the difference of terms (4) and (5), their comparison allows us to evaluate the relationship between social influence and self-regulation by means of self-regulation strength ${\beta }_{v}$ and degree ${k}_{v}$ of player $v$. We prove the following result.

**Main result 1**. *If for each player self-regulation is stronger than connectivity*, ${\beta }_{v} > {k}_{v}$*, then the fully cooperative configuration* ${{\bf{x}}}_{ALLC}^{\ast }={[1,1,\ldots ,1]}^{\top }$ *is an attractor for the system dynamics, while at the same time total defection* ${{\bf{x}}}_{ALLD}^{\ast }={[0,0,\ldots ,0]}^{\top }$ *is repulsive*.

These stability and instability properties of ${{\bf{x}}}_{ALLC}^{\ast }$ and ${{\bf{x}}}_{ALLD}^{\ast }$ have been formally proved by using linear stability theory (see Theorems 3 and 4 of the SI document). Stronger results highlight the relationship among global stability of ${{\bf{x}}}_{ALLC}^{\ast }$, initial conditions, and payoffs $T > 1$ and $S < 0$. To this aim, it is useful to introduce the parameter $\rho \ge 1$:

7 $$\rho =\frac{max\{T-1,-S\}}{min\{T-1,-S\}}.$$

When the effect of $T$ is enough stronger than $S$, the game is driven by the temptation to defect (*T-driven*). Accordingly, in this case $\rho =(1-T)/S$. On the other hand, when the effect of $S$ is stronger than $T$, then the game is driven by the fear to be betrayed (*S-driven*). In this case, $\rho =S/(1-T)$. In both *T-driven* and *S-driven* cases, $\rho \ge 1$. Further details can be found in the SI document. The parameter $\rho$ is involved in the following results concerning the global stability of ${{\bf{x}}}_{ALLC}^{\ast }$ and ${{\bf{x}}}_{ALLD}^{\ast }$. The proofs, based on finding suitable nonlinear functions satisfying the Lyapunov stability theory, are given in Theorems 5 and 6 of the SI document.

 **Main result 2**. *If, for each player* $v$, ${\beta }_{v} > {k}_{v}\rho$ *, then* ${{\bf{x}}}_{ALLC}^{\ast }$ *is a global attractor for any initial strategy* ${x}_{v}(0)\in (0,1]$*, thus all members of the population will be eventually cooperators*.

 **Main result 3**. *If, for each player* $v$ , ${\beta }_{v} < {k}_{v}/\rho$*, then* ${{\bf{x}}}_{ALLD}^{\ast }$ *is a global attractor for any initial strategy* ${x}_{v}(0)\in [0,1)$*, thus all members of the population will be eventually defectors*.

Therefore, under stronger conditions than the one assumed in Main result 1, ${{\bf{x}}}_{ALLC}^{\ast }$ is globally attractive and ${{\bf{x}}}_{ALLD}^{\ast }$ is globally repulsive.

The previous results are related to the global emergence of cooperation, arising when all members of a networked population turn their strategies to cooperation. However, cooperation can partially emerge; for example, some players may exhibit partial levels of cooperation.

Let ${\mathscr{C}}=\{w:{\beta }_{w} > {k}_{w}\rho \}$ be the set of players which satisfy Main result 2, thus, if $v\in {\mathscr{C}}$, then ${x}_{v}(+\infty )=1$ for any initial condition ${x}_{v}(0)\in (0,1]$. Let ${\mathscr{D}}=\{w:0\le {\beta }_{w} < {k}_{w}/\rho \}$ be the set of players satisfying Main result 3. If $v\in {\mathscr{D}}$, then ${x}_{v}(+\infty )=0$, independently on the initial condition ${x}_{v}(0)\in [0,1)$. The dynamics of individuals in both these sets are also independent on the behavior of any other player of the population.

Interestingly, a richer set of unexpected behaviors is observed for the *uncertain players*, belonging to the set ${\mathscr{U}}=\{w:{k}_{w}/\rho \le {\beta }_{w}\le {k}_{w}\rho \}$, for which it is not guaranteed that ${x}_{v}(+\infty )=1$, nor ${x}_{v}(+\infty )=0$. The dynamics of these individuals has been investigated by means of extensive numerical experiments.

**Figure 2:**
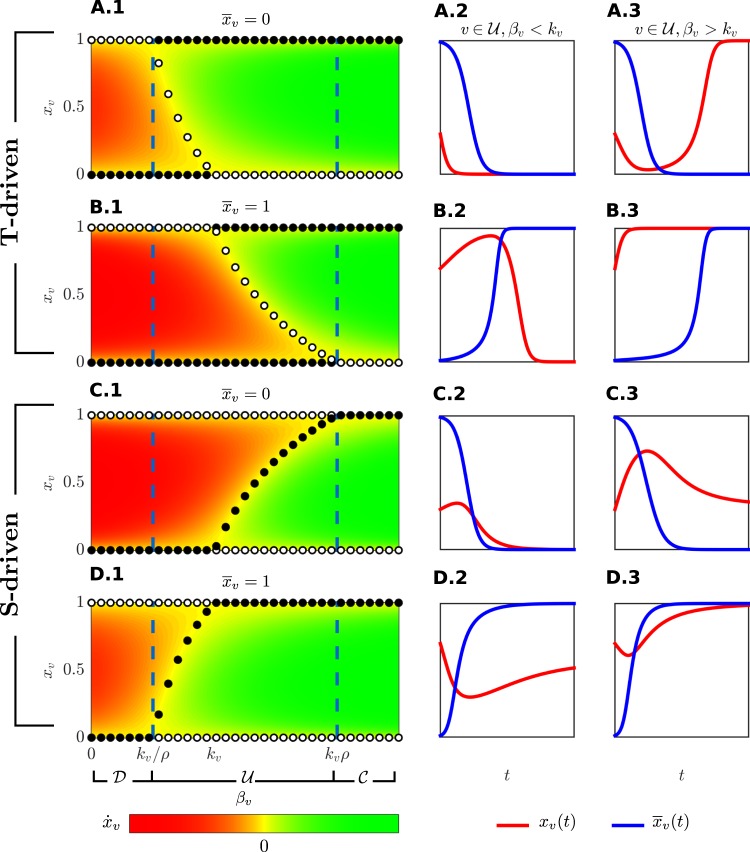
Flow and dynamics. The value of the derivative ${\dot{x}}_{v}$ is plotted as a function of ${x}_{v}$ and ${\beta }_{v}$, with ${k}_{v}=10$, together with attractive (black) and repulsive (white) steady states. For a *T-driven* game ($T=3$, $S=-1$ and $\rho =2$), the time derivatives of ${x}_{v}$ for a generic player connected only to full defectors (${\bar{x}}_{v}=0$) and only to full cooperators (${\bar{x}}_{v}=1$) are shown in (**A.1**,**B.1**), respectively. Similarly, (**C.1**,**D.1**) show the time derivatives of ${x}_{v}$ for a *S-driven* game ($T=2$, $S=-2$ and $\rho =2$), assuming a neighborhood of full defectors and full cooperators, respectively. Vertical dashed lines are drawn for ${\beta }_{v}={k}_{v}/\rho$ and ${\beta }_{v}={k}_{v}\rho$, thus separating the regions ${\mathscr{D}}$, ${\mathscr{U}}$ and ${\mathscr{C}}$. Some examples of the time courses of ${x}_{v}(t)$ (red) and of ${\bar{x}}_{v}(t)$ (blue) for a player $v\in {\mathscr{U}}$ are depicted in **A.2**, **B.2**, **C.2** and **D.2** for ${\beta }_{v} < {k}_{v}$, and in **A.3**, **B.3**, **C.3** and **D.3** for ${\beta }_{v} > {k}_{v}$.

First of all, in Fig. 2 some prototypical examples of the asymptotic dynamics and the corresponding flows of uncertain players are reported. The solutions have been obtained by solving the ODE system (6) through an explicit Runge-Kutta (4,5) formula^55^. This numerical method has been used for all simulations reported in this paper. In the subplots 2A.1, 2B.1, 2C.1 and 2D.1, assuming that the equivalent player reached a fully cooperative (${\bar{x}}_{v}=1$) or fully defective (${\bar{x}}_{v}=0$) steady state, and for a constant value of ${k}_{v}$, the value of ${\dot{x}}_{v}$ is drawn with different colors, according to the values of ${\beta }_{v}$ and ${x}_{v}$. In the same figures, the attracting (black circles) and repulsive (white circles) steady states of ${x}_{v}$ are depicted. One can notice that in a *T-driven* prisoner’s dilemma game (subplots 2A.1 and 2B.1) there are repulsive partially cooperative equilibria ${x}_{v}^{\ast }$. These equilibria separate the phase space as thresholds, thus giving rise to a bistable dynamics leading player to full cooperation or full defection, for any initial condition ${x}_{v}(0)\in (0,1)\backslash \{{x}_{v}^{\ast }\}$. Existence and feasibility of partially cooperative equilibria are discussed in Theorems 1 and 2 of the SI document. Moreover, by increasing ${\bar{x}}_{v}$ from 0 to 1, the green area reduces, thus decreasing the probability for individuals to be cooperative.

The time courses ${x}_{v}$ of player $v$ (red line) interacting with unconditional defective or unconditional cooperative equivalent players ${\bar{x}}_{v}$ (blue line), are depicted in the second and third columns of Fig. 2, where, for example, we observe the onset of reciprocity mechanisms, for which $v$ defects if his neighborhood defects (subplot 2A.2) or $v$ cooperates if his neighborhood cooperates (subplot 2B.3). Interestingly, also anti-reciprocal behaviors arise: $v$ may cooperate if others defect, as shown in subplot 2A.3. In this case, the absence of cooperators in the neighborhood to be exploited, makes $v$ aware on the importance of being cooperative. On the contrary, the abundance of cooperators in the neighborhood may lead player $v$ towards defection, in order to exploit nearby players (subplot 2B.2).

For the *S-driven* games (subplots 2C.1 and 2D.1), the partially cooperative steady states ${x}_{v}^{\ast }$ are attractive, thus ensuring that players $v\in {\mathscr{U}}$ will reach at least a partial level of cooperation. Interestingly, since the punishment effect is strong, the presence of cooperators in the neighborhood facilitates the convergence to a cooperative state. Specifically, if ${\bar{x}}_{v}$ moves from 0 (subplot 2C.1) to 1 (subplot 2D.1), the probability for a player to be cooperative increases. Subplots 2C.2–2C.3, and 2D.2–2D.3 depict some examples of the time course of the solutions when playing against unconditional defective or unconditional cooperative equivalent players. Reciprocity mechanisms are observed also for the *S-driven* case, for example, defector-defector (subplot 2C.2), partially cooperator-cooperator (subplot 2D.2), cooperator-cooperator (subplot 2D.3). Finally, Fig. 2C.3 shows an anti-reciprocal behavior, where $v$ partially cooperates when the neighborhood defects.

**Figure 3:**
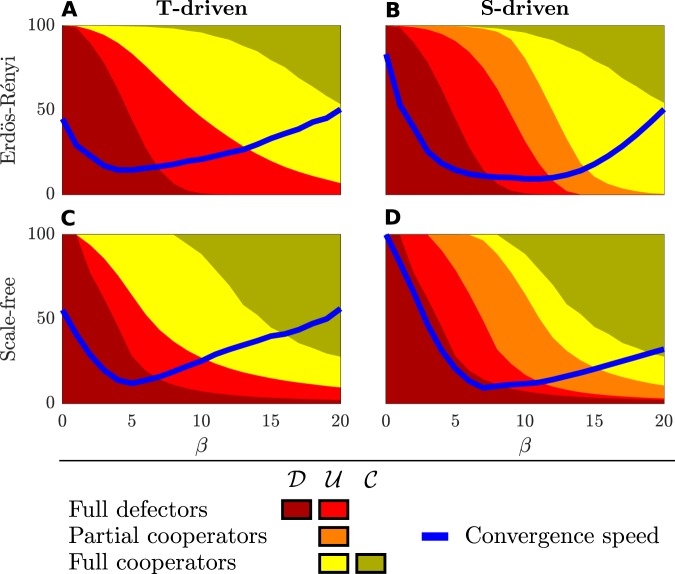
Average distribution of strategies and convergence speed. Four different setups are considered: Erdös-Rényi for *T-driven* (**A**) and *S-driven* (**B**) games, and Scale-Free for *T-driven* (**C**) and *S-driven* (**D**) cases. 500 graphs with $N=1000$ nodes and average degree $\bar{k}=10$ have been generated for each topology. For different values of the parameter ${\beta }_{v}=\beta \in \{0,\ldots ,20\}$ and using random initial conditions in the set $(0,1)$, the SR-EGN equation is simulated until a steady state is reached. The values of $T$ and $S$ are the same as in Fig. 2. The average distribution of strategies of the whole population is shown for defectors (red), partial cooperators (orange), and cooperators (yellow). The blue lines represent the convergence speed, experimentally estimated as the inverse of the time required to reach the steady state.

In addition to the local dynamics of players, the global behavior of the system is studied by investigating the distribution of cooperation over the whole population. To this aim, extensive simulations have been run using random networks with Erdös-Rényi and Scale-Free degree distributions. For each model, 500 networks of $N=1000$ nodes have been generated, with average degree $\bar{k}=10$. The the SR-EGN equation has been simulated with random initial conditions, assuming that all individuals share the same self-regulating factor ${\beta }_{v}=\beta$. In Fig. 3 the resulting percentages of full defectors (red areas), partial cooperators (orange areas) and full cooperators (yellow areas) at steady state are reported for both the *T-driven* and *S-driven* games and different values of $\beta \in \{0,\ldots ,20\}$. Color intensities are used to distinguish the sets ${\mathscr{D}},{\mathscr{U}}$ and ${\mathscr{C}}$: dark red areas indicate the individuals in class ${\mathscr{D}}$ and dark yellow the individuals in class ${\mathscr{C}}$. As expected from the theoretical results, increasing $\beta$ produces an increase of the number of cooperators.

Anyway, Fig. 3 clearly shows that uncertain individuals $v\in {\mathscr{U}}$ are also able to cooperate (light yellow areas), partially cooperate in the *S-driven* game (orange areas in subplots 3B and 3D) or defect (light red areas), thus showing that cooperation is possible also for lower values of $\beta$. This is very important in real applications, because the high values of $\beta$ required by the theoretical results are usually associated to high costs for the individuals.

Finally, we notice that this phenomenon is stronger for the Scale-Free than for the Erdös-Rényi networks, thus suggesting that the heterogeneity of a network is highly effective in promoting cooperation. Furthermore, the average convergence speed of the system to a steady state, reported by blue lines in Fig. 3, changes with $\beta$. In particular, it decreases for small $\beta$s, due to the transition from all defectors to a mixed situation, where cooperators and defectors coexist. Finally, when the number of cooperators dominate the population, the speed increases again.

### Game transitions

A significant result of this study is that self-regulation induces game transitions, resulting from changes in the structure of the underlying games. To explain this phenomenon, we calculate the equivalent game of player $v$ and we find that parameter $\rho$, player’s degree ${k}_{v}$, self-regulating strength ${\beta }_{v}$, as well as the strategy of the equivalent player ${\bar{x}}_{v}$, are responsible for these transitions (see the SI document for technical details).

A short recap on two strategy games is useful. Four game typologies are possible: PD game, where defection is the only dominant strategy; Stag Hunt game (SH), where cooperation (defection) is the best response to cooperation (defection); Chicken game (CH), where cooperation is the best response to defection, and vice versa; Harmony game (HA), where cooperation is the only dominant strategy.

In the SR-EGN equation, player $v$ is an unconditional defector when ${\beta }_{v} < {k}_{v}/\rho$. Indeed he belongs to ${\mathscr{D}}$ and his equivalent game is still a PD. On the other hand, player $v$ is an unconditional cooperator when ${\beta }_{v} > {k}_{v}\rho$. In this case, he belongs to ${\mathscr{C}}$, and the equivalent game played by player $v$ is now HA. Notice that in both situations, the equivalent game does not depend on the behavior of the equivalent player ${\bar{x}}_{v}$.

In the intermediate region ${\mathscr{U}}$, the dynamics of player $v$ is uncertain, and transitions may dynamically occur since the equivalent game is also influenced by the behavior of the neighboring players. In the *T-driven* case, when ${k}_{v}/\rho  < {\beta }_{v} < {k}_{v}$, as ${\bar{x}}_{v}$ decreases, the temptation of $v$ to defect is reduced, and thus transitions from PD to SH games are observed. Instead, for increasing values of ${\bar{x}}_{v}$, transitions from SH to PD occur. Similarly, when ${k}_{v} < {\beta }_{v} < {k}_{v}\rho$, as ${\bar{x}}_{v}$ decreases, the fear of player $v$ to be betrayed is reduced, thus transitions from SH to HA games are observed. Conversely, for increasing values of ${\bar{x}}_{v}$, transitions from HA to SH arise. For the *S-driven* case, when ${k}_{v}/\rho  < {\beta }_{v} < {k}_{v}$, the observed transitions are from PD to CH games when the equivalent player ${\bar{x}}_{v}$ increases his cooperation. Indeed, this raise is able to inhibit the player $v$’s fear to be betrayed. Finally, when ${k}_{v} < {\beta }_{v} < {k}_{v}\rho$, $v$ moves from a CH to a HA game as ${\bar{x}}_{v}$ increases; the transition is due to the inhibition of the temptation of $v$ to defect. All the described phenomena dynamically occur, provided that cooperation ${\bar{x}}_{v}$ varies enough.

**Figure 4:**
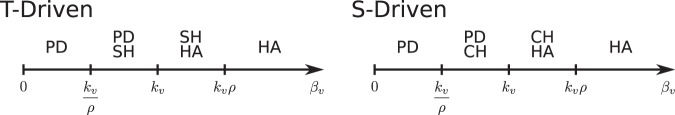
SR-EGN equation: schematic representation of game transitions.

These results are summarized schematically in Fig. 4, confirming the anti-reciprocal (reciprocal) behaviors already discussed for the *T-driven* (*S-driven*) case.

Additionally, we remark that the parameter $\rho$ plays a role for the emergence of cooperation in the group of the uncertain players. Indeed, the size ${k}_{v}(\rho -1/\rho )$ of the interval ${{\mathscr{U}}}_{v}=\{{\beta }_{v}:v\in {\mathscr{U}}\}=[{k}_{v}/\rho ,{k}_{v}\rho ]$ is larger as $\rho$ increases, thus favoring the onset of game transitions and concurring to explain better the results shown in Fig. 3, for which the cooperation depends mainly on uncertain players.

### The role of network structure

**Figure 5:**
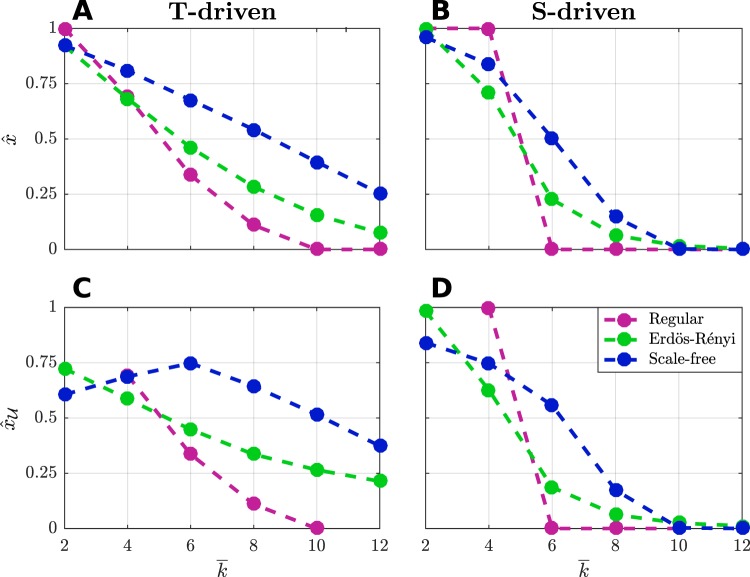
Average cooperation vs. average degree. The average cooperation level $\widehat{x}$ of the whole population at steady state is reported for *T-driven* (**A**) and *S-driven* (**B**) games as a function of the average degree $\bar{k}\in \{2,4,\ldots ,12\}$. The population is composed by $N=1000$ players and it is organized over regular (magenta), Erdös-Rényi (green) and Scale-Free (blue) random networks. Similarly, in (**C**,**D**) the average cooperation ${\widehat{x}}_{{\mathscr{U}}}$ of the subpopulation of players $v\in {\mathscr{U}}$, is depicted. The values have been averaged over 500 simulations for each network topology and for each game. In all cases, ${\beta }_{v}=\beta =5$. The values of $T$ and $S$ are the same as in Fig. 2.

As aforementioned, the network structure plays a significant role for the emergence of cooperation. Fig. 5 shows the average cooperation of the whole population, calculated as $\widehat{x}=\frac{1}{NI}{\sum }_{i=1}^{I}{\sum }_{v=1}^{N}{x}_{v}(\infty )$, where $I=500$ is the number of random instances of the experiment, which include network generation and initial conditions. In all simulations, ${\beta }_{v}=\beta =5$. The behavior of $\widehat{x}$ is reported in the subplots 5A (*T-driven*) and 5B (*S-driven*), by varying $\bar{k}$ in $\{2,4,\ldots ,12\}$, for regular (magenta), Erdös-Rényi (green) and Scale-Free (blue) networks. Subplots 5C and 5D report the average cooperation ${\widehat{x}}_{{\mathscr{U}}}$ of the subpopulation of uncertain players $v\in {\mathscr{U}}$.

Interestingly, except for very small values of $\bar{k}$, random networks, and especially the Scale-Free networks, foster cooperation more than regular ones. This fact is more relevant for the subset of uncertain players (subplots 5C and 5D), suggesting that these players have a crucial role for cooperation.

The presented model allows to study the dynamics of every member of the population, thus enabling to observe whether an individual is changing opinion over time. Moreover, we are interested in understanding the relationship between the dynamics of each individual and that of his equivalent player, thus highlighting different possible behaviors, such as mutual defection, recruiting (cooperate if others cooperate), exploitation of cooperators (defect if others cooperate) and altruism (cooperate if others defect). In order to quantify the difference between the level of cooperation of player $v$ and the average cooperation of his neighbors at steady state, the following indicator is introduced:

8 $${c}_{v}={x}_{v}(\infty )-{\bar{x}}_{v}(\infty ).$$

If ${c}_{v}\simeq 0$, the player exhibits mutual behavior. On the contrary, for ${c}_{v}$ sufficiently different from 0, we observe opposite behaviors. Specifically, if ${c}_{v} > 0$, then player $v$ shows altruism, since his level of cooperation is higher than the average of his neighbors, while ${c}_{v} < 0$ indicates exploitation of cooperators.

**Figure 6:**
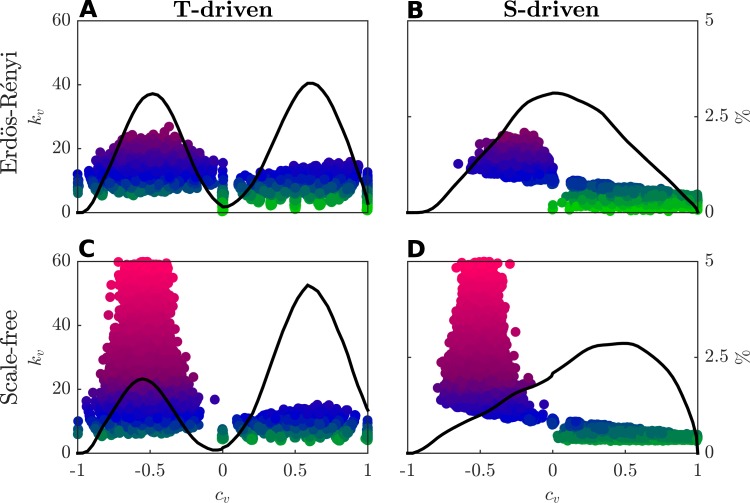
Selfishness and altruism within heterogeneous populations. Using the same experimental setup developed for Fig. 3, for each player, we report a dot representing the value ${c}_{v}$ ($x$-axis) and his degree ${k}_{v}$ ($y$-axis). The color of each dot indicates the degree ${k}_{v}$ of player $v$, thus allowing to distinguish among classes ${\mathscr{C}}$ (green dots), ${\mathscr{U}}$ (blue dots) and ${\mathscr{D}}$ (magenta dots). The self-regulation parameter $\beta$ is set to 10 for all players. The black lines represent the distribution ($\%$) of the indicator ${c}_{v}$ over the whole population.

Under the same experimental setup used for Fig. 5, Fig. 6 reports a dot for each player, representing the combination of the value ${c}_{v}$ ($x$-axis) and his degree ${k}_{v}$ ($y$-axis). Moreover, the color of each dot corresponds to the degree ${k}_{v}$ of player $v$, thus allowing to distinguish between cooperative players in class ${\mathscr{C}}$ (green dots), uncertain players in class ${\mathscr{U}}$ (blue dots) and defective players in class ${\mathscr{D}}$ (magenta dots). The self-regulation parameter $\beta$ is set to 10 for all players. The black lines in Fig. 6 represent the distribution over the whole population of the indicator ${c}_{v}$. Players exhibiting mutual behavior are mainly present in the *S-driven* games, as reported by black lines in subplots 6B and 6D. In the *T-driven* case, the population mainly shows oppositing behaviors, and it is split into two groups composed by altruistic and selfish players, respectively (subplots 6A and 6C). It is worthwhile to notice that altruistic players (${c}_{v} > 0$) belong to classes ${\mathscr{C}}$ and ${\mathscr{U}}$, thus showing a low/intermediate level of connectivity within the network, while selfish players (${c}_{v} < 0$) belong to class ${\mathscr{D}}$, where the connectivity is high. Moreover, while the distribution of ${c}_{v}$ values is symmetric with respect to 0 for the Erdös-Rényi networks, as shown in subplots 6A and 6B, it is asymmetric when the network of connections is Scale-free, as reported in subplots 6C and 6D. In fact, Scale-free networks include a higher number of altruistic players as they present a high number of lowly connected players, which concur to activate reciprocal mechanisms.

Joining the results of Figs. 2, 3, 5 and 6, we conclude that some individuals are more sensitive and aware of their internal mechanisms, thus becoming cooperative for lower self-regulating factors, and exhibiting a more altruistic behavior. In particular, for the *S-driven* game, these receptive individuals catalyze the others to cooperate.

## Discussion

This paper proposes the analysis of the interplay between social influence and self-regulating mechanisms in continuous models describing the strategic interactions among the members of a networked population. The EGN equation has been appropriately extended to account for a self-regulation feedback, thus giving rise to the SR-EGN equation, which activates stable processes opposing the natural tendency towards defection, typical of the prisoner’s dilemma game. Theoretical results ensure that cooperation globally emerges in the extended model, whether self-regulation is stronger than social pressure. Similarly, low self-regulation will let defection spreading out all over the population.

The theoretical results presented in this study are based on the stability analysis of steady states, representing the full or partial cooperative or defective asymptotic behavior of the individuals. The time required for the individuals to reach cooperative states has been also investigated.

From a practical perspective, we found that for intermediate levels of self-regulation, cooperation may partially emerge as the result of different mechanisms: cooperative reciprocity, which activates a recruiting process, and cooperative anti-reciprocity (altruism) arising from the awareness of individuals. These results are coherent with the occurrence of game transitions. Numerical simulations show that the recruiting process is mostly driven by lowly connected neighbors, while awareness mechanisms are prominently caused by highly connected neighbors. Coherently, we find that the Scale-Free topologies are the most cooperative. Indeed, on one hand, they naturally present a high number of lowly connected players, which concur to activate reciprocal mechanisms, while the typical presence of highly connected players (hubs) activates the anti-reciprocal mechanisms. On the other hand, the Erdös-Rényi topologies are less cooperative since they present approximately the same number of lowly and highly connected neighbors. Additionally, the regular networks are in general the least cooperative since the distinction between lowly and highly connected players vanishes. Hence, it can be concluded that cooperation is more likely in heterogeneous network structures.

We want also to highlight that the presence of self-regulation mechanisms entails some costs, which can be reasonably proportional to parameter $\beta$. This means that a policy based on the theoretical results reported in this work guarantees that a certain amount of people will unconditionally cooperate, and that, at the same time, they will produce higher costs for the society. The good news is that the same level of cooperation can be achieved by using lower values of $\beta$, relying on the spontaneous emerging and learning processes of recruiting and awareness, thus reducing significantly the social impact. Heterogeneous populations connected by a Scale-Free connection structure are able to additionally foster the emergence of this “low-cost” cooperation.

In the present work a homogeneous assignment of game parameters $T$ and $S$ to each member of the population is assumed. This choice is motivated for easing the understanding of the self-regulating mechanisms. Anyway, the SR-EGN equation naturally incorporates the possibility of assuming more heterogeneous setup, such as opponent-specific payoffs, as well as different self-game structures. Future efforts will be devoted to investigate these cases.

## Supplementary information

Supplementary information
