# Supplementary material for: Self-regulation versus social influence for promoting cooperation on networks
Source: Sci Rep. 2020 Mar 16;10:4830. doi: 10.1038/s41598-020-61634-7 (PMC7075901; doi:10.1038/s41598-020-61634-7)
Supplement: Supplementary file 1 — Supplementary information [file 41598_2020_61634_MOESM1_ESM.pdf]

Self-regulation versus social influence for promoting cooperation on networks

Supporting information document

Dario Madeo[1], Chiara Mocenni[1]

[1]Department of Information Engineering and Mathematics, University of Siena.

1 Preliminaries

1.1 The Evolutionary Game equation on Networks (EGN) and self games

Let $\mathcal{V} = \{1, 2, \ldots, N\}$ be the set of players. Each player is placed in a vertex of an undirected graph, defined by the adjacency matrix $\mathbf{A} = \{a_{v,w}\} \in \{0, 1\}^{N\times N}$ with $(v, w) \in \mathcal{V}^2$. Specifically, $a_{v,w} = 1$ when v is connected to w, 0 otherwise. It is also assumed that $a_{v,v} = 0$. The degree of player v is defined as the cardinality of his neighborhood, namely:

$$k_v = \sum_{w=1}^{N} a_{v,w}.$$

In the literature on evolutionary game theory, it is assumed that, at each time, one individual uses a pure strategy in a given set while playing games with connected individuals. These games are often modeled as Prisoner's dilemma games, where the set of pure strategies contains only two elements: cooperation (C) and defection (D). The outcome of those games is described by the following payoff matrix:

$$\mathbf{B} = \begin{bmatrix} R & S \\ T & P \end{bmatrix},$$

where R is the reward when both players cooperate, T is the temptation to defect when the opponent cooperates, S is the sucker's payoff earned by a cooperative player when the opponent defects, and P is the punishment for mutual defection. More specifically, for a Prisoner's dilemma game, the reward is a better outcome than the punishment ($R > P$), the temptation payoff is higher than the reward ($T > R$), and the punishment is preferred to the sucker's payoff ($P > S$). Without loss of generality, we assume $R = 1$ and $P = 0$, thereby normalizing the advantage of mutual cooperation over mutual defection to 1 [S1]. Under this assumption, $T > 1$ and $S < 0$. It is straightforward to note that $T - 1$ quantifies the temptation to defect, while $-S$ is related to the disadvantage of being defected. Therefore, we can distinguish two cases:

- If $T - 1 > -S$, then the temptation to defect is stronger than the disadvantage of being defected. This game will be hereafter called *T-driven*.
- If $T - 1 < -S$, then the disadvantage of being defected is stronger than the temptation to defect. This game will be hereafter called *S-driven*.

At each time instant, an individual v will play k_v continuous prisoner's dilemma games with his neighbors [S2, S3, S4]; specifically, he chooses his own level of cooperation indicated by $x_v \in [0,1]$. Notice that discrete strategies C and D are special cases obtained for $x_v = 1$ and $x_v = 0$, respectively. When any two connected players v and w take part in a game, the payoff for v is defined by the continuous function $\phi : [0,1] \times [0,1] \to \mathbb{R}$ (see [S4]):

$$\begin{aligned}\phi(x_v, x_w) &= (R - T + P - S)x_v x_w + (S - P)x_v + (T - P)x_w + P \\ &= (1 - T - S)x_v x_w + S x_v + T x_w. \end{aligned} \tag{1}$$

The total payoff ϕ_v of player v is the sum of all outcomes of two-player games with neighbors. Formally, the payoff function $\phi_v : [0,1]^N \to \mathbb{R}$ is defined as follows:

$$\phi_v(\mathbf{x}) = \sum_{w=1}^{N} a_{v,w}\phi(x_v, x_w),$$

where $\mathbf{x}$ is the vector of all the x_v variables. Moreover, given the vector $\mathbf{x}$, we define the following payoff of pure strategies C ($x_v = 1$) and D ($x_v = 0$):

$$\begin{cases} p_v^C(\mathbf{x}) = \sum_{w=1}^{N} a_{v,w}\phi(1, x_w) = \sum_{w=1}^{N} a_{v,w}[(R - S)x_w + S] = \sum_{w=1}^{N} a_{v,w}[(1 - S)x_w + S] \\ p_v^D(\mathbf{x}) = \sum_{w=1}^{N} a_{v,w}\phi(0, x_w) = \sum_{w=1}^{N} a_{v,w}[(T - P)x_w + P] = \sum_{w=1}^{N} a_{v,w} T x_w \end{cases}.$$

Following [S5, S6], the EGN equation for two-strategy games reads as follows:

$$\dot{x}_v = x_v(1 - x_v)\Delta p_v(\mathbf{x}), \tag{2}$$

where

$$\Delta p_v(\mathbf{x}) = p_v^C(\mathbf{x}) - p_v^D(\mathbf{x}) = \sum_{w=1}^{N} a_{v,w}[(1 - T - S)x_w + S].$$

It is clear that the level of cooperation of player v increases (decreases) when $\Delta p_v(\mathbf{x})$ is positive (negative). In other words, the player v will be more cooperative over time as long as the payoff he can earn using the pure strategy C is better than the payoff he can earn using the pure strategy D.

This evaluation of the benefits provided by the available strategies can be formulated in an alternative way. Specifically, suppose that player v is able to appraise whether a change of his strategy x_v produces an improvement of his payoff ϕ_v. This means that, if the derivative of ϕ_v with respect to x_v is positive (negative), the player would like to increase (decrease) his level of cooperation. According to this idea, notice that:

$$\begin{aligned}\frac{\partial \phi_v(\mathbf{x})}{\partial x_v} &= \sum_{w=1}^{N} a_{v,w}\frac{\partial \phi(x_v, x_w)}{\partial x_v} \\ &= \sum_{w=1}^{N} a_{v,w}[(1 - T - S)x_w + S] \\ &= \Delta p_v(\mathbf{x}).\end{aligned}$$

Thus, the EGN equation (2) can be rewritten as follows:

$$\dot{x}_v = x_v(1 - x_v)\frac{\partial \phi_v(\mathbf{x})}{\partial x_v}. \tag{3}$$

Since in (3) $x_v(1-x_v) \geq 0$, then the sign of $\dot{x}_v$ depends only on the term $\partial\phi_v/\partial x_v$, which involves the states x_w of all neighbors, rather than the current state x_v of player v himself. Then, if this term is positive (negative), player v would like to increase (decrease) his level of cooperation x_v. Of course, when it is null, player v has no incentives to change his mind.

It is worthwhile to notice that, while the replicator equation is used to describe the dynamics of population where strategies correspond to the phenotypes of the individuals, its extension on graphs, the EGN equation, is suitable for analyzing the dynamics of individuals arranged on a network, and able to choose their strategies in the continuous set $[0, 1]$.

The EGN equation (3), as well as most of the models presented in the literature, assumes that the strategy dynamics of a generic player v is driven only by external factors. Indeed, $\frac{\partial\phi_v}{\partial x_v}$ depends only on the state of neighboring players, not on the current state x_v of player v himself. Inspired by mechanisms describing self-regulation in animal societies reported in [S7], we overcome this issue by introducing the Self-Regulated EGN equation (SR-EGN); this new model is obtained by adding a self-regulating term f_v to the EGN equation, balancing the external feedback $\frac{\partial\phi_v}{\partial x_v}$. The SR-EGN equation reads as:

$$\dot{x}_v = x_v(1-x_v)\left(\frac{\partial\phi_v(\mathbf{x})}{\partial x_v} - \beta_v f_v(x_v)\right), \tag{4}$$

where the parameter β_v is used to tune the effectiveness of the introduced self-regulation mechanism. Specifically, we assume that this self-regulation term embodies a game that a given individual plays against himself. To describe this self game, consider two generic players, choosing feasible strategies y and z. As already mentioned, the first player can assess whether a change of his strategy y can lead to an improvement of the payoff $\phi(y, z)$. In particular, the assessment is based on the sign of the partial derivative

$$\frac{\partial\phi(y,z)}{\partial y} = (1-T-S)z + S.$$

In the particular case of individuals representing both the first and second player at the same time, the derivative reads as follows:

$$\left.\frac{\partial\phi(y,z)}{\partial y}\right|_{y=x_v, z=x_v} = (1-T-S)x_v + S.$$

Therefore, the self-regulating term is defined as:

$$f_v(x_v) = \left.\frac{\partial\phi(y,z)}{\partial y}\right|_{y=x_v, z=x_v} = (1-T-S)x_v + S.$$

Notice that function $f_v(x_v)$ in equation (4) depends on x_v. Thus, the self game introduces a **feedback** mechanism regulated by the parameter $\beta_v \in \mathbb{R}$. In particular, in equation (4), $\beta_v > 0$ represents a negative feedback, $\beta_v < 0$ stands for a positive feedback, while $\beta_v = 0$ refers to situations where the player v does not play a self game.

1.2 Steady states and linearization

A steady state $\mathbf{x}^*$ is a solution of equation (4) satisfying $\dot{x}_v = 0\ \forall v \in \mathcal{V}$. In order to be **feasible**, the components of a steady state must belong to the set $[0, 1]$. Formally, the set of feasible steady states is:

$$\Theta = \{\mathbf{x}^* \in \mathbb{R}^N : \dot{x}^*_v = 0 \wedge x^*_v \in [0, 1]\ \forall v \in \mathcal{V}\}.$$

It is clear that all points such that for all v, $x^*_v = 0$ or $x^*_v = 1$ are in the set Θ. We remark that set Θ may contain also other steady states, exhibiting partial levels of cooperation. Among the pure steady states, particularly relevant are the followings:

$$\mathbf{x}^*_{ALLC} = [1, 1, \ldots, 1]^\top,$$

and

$$\mathbf{x}^*_{ALLD} = [0, 0, \ldots, 0]^\top.$$

Indeed, they represent a population composed by full cooperators and full defectors, respectively, and thus they describe the spread of cooperation, or alternatively its extinction in a given population.

The dynamical properties of these two pure steady states is fundamental for the emergence of cooperation. In particular, their stability can be locally analyzed by linearizing system (4).

The Jacobian matrix of system (3), $\mathbf{J}(\mathbf{x}) = \{j_{v,w}(\mathbf{x})\}$, is defined as follows:

$$j_{v,w}(\mathbf{x}) = \frac{\partial \dot{x}_v}{\partial x_w} = \begin{cases} x_v(1-x_v)(1-T-S), & \text{if } a_{v,w} = 1 \\ (1-2x_v)\left(\frac{\partial \phi_v(\mathbf{x})}{\partial x_v} - \beta_v f_v(x_v)\right) - \beta_v x_v(1-x_v)(1-T-S), & \text{if } w = v \\ 0, & \text{otherwise} \end{cases}.$$

It is easy to show that the Jacobian matrix reduces to a diagonal one for both $\mathbf{x}^*_{ALLC}$ and $\mathbf{x}^*_{ALLD}$. Moreover, observe that:

$$\begin{aligned} \left.\frac{\partial \phi_v(\mathbf{x})}{\partial x_v}\right|_{\mathbf{x}^*_{ALLC}} &= \sum_{w=1}^{N} a_{v,w}[(1-T-S)\cdot 1 + S] = \sum_{w=1}^{N} a_{v,w}(1-T) = k_v(1-T), \\ \left.\frac{\partial \phi_v(\mathbf{x})}{\partial x_v}\right|_{\mathbf{x}^*_{ALLD}} &= \sum_{w=1}^{N} a_{v,w}[(1-T-S)\cdot 0 + S] = \sum_{w=1}^{N} a_{v,w}S = k_v S, \\ f_v(1) &= (1-T-S)\cdot 1 + S = 1-T, \\ f_v(0) &= (1-T-S)\cdot 0 + S = S. \end{aligned}$$

Therefore:

$$j_{v,v}(\mathbf{x}^*_{ALLC}) = (1 - 2\cdot 1)\left(\left.\frac{\partial \phi_v(\mathbf{x})}{\partial x_v}\right|_{\mathbf{x}^*_{ALLC}} - \beta_v f_v(1)\right) = (T-1)(k_v - \beta_v)$$

for $\mathbf{x}^*_{ALLC}$, and

$$j_{v,v}(\mathbf{x}^*_{ALLD}) = (1 - 2\cdot 0)\left(\left.\frac{\partial \phi_v(\mathbf{x})}{\partial x_v}\right|_{\mathbf{x}^*_{ALLD}} - \beta_v f_v(0)\right) = S(k_v - \beta_v)$$

for $\mathbf{x}^*_{ALLD}$.

The system (4) may have steady states with some components in the set $(0, 1)$. Consider a steady state $\mathbf{x}^*$ and suppose that one component x^*_v belongs to the set $(0, 1)$. Therefore:

$$\left.\left(\frac{\partial \phi_v(\mathbf{x})}{\partial x_v} - \beta_v f_v(x_v)\right)\right|_{\mathbf{x}=\mathbf{x}^*} = 0. \tag{5}$$

Notice that:

$$\begin{aligned}\frac{\partial \phi_v(\mathbf{x})}{\partial x_v} - \beta_v f_v(x_v) &= \sum_{w=1}^{N} a_{v,w}[(1-T-S)x_w + S] - \beta_v\left((1-T-S)x_v + S\right) \\ . &= (1-T-S)\sum_{w=1}^{N} a_{v,w}x_w + \sum_{w=1}^{N} S - \beta_v\left((1-T-S)x_v + S\right) \\ &= k_v(1-T-S)\frac{1}{k_v}\sum_{w=1}^{N} a_{v,w}x_w + k_v S - \beta_v\left((1-T-S)x_v + S\right) \\ &= k_v[(1-T-S)\overline{x}_v + S] - \beta_v[(1-T-S)x_v + S], \qquad (6)\end{aligned}$$

where

$$\overline{x}_v = \frac{1}{k_v}\sum_{w=1}^{N} a_{v,w}x_w,$$

represents an *equivalent player*, incorporating the average decisions of all neighbors of player v.

For $\mathbf{x} = \mathbf{x}^*$, we have that equation (5) can be rewritten as:

$$k_v[(1-T-S)\overline{x}_v^* + S] - \beta_v[(1-T-S)x_v^* + S = 0,$$

yielding to

$$x_v^* = \frac{k_v}{\beta_v}\overline{x}_v^* - \frac{S}{1-T-S}\left(1 - \frac{k_v}{\beta_v}\right).$$

The following theoretical results on the feasibility of x_v^* hold.

Theorem 1. *Consider an T-driven game. Let* $\rho = \frac{1-T}{S}$ *and let* $Q_v = \frac{(\rho-1)k_v\overline{x}_v^* + k_v}{\rho}$. *Then*

$$\beta_v \in (Q_v, \rho Q_v) \iff x_v^* \in (0,1).$$

Proof. First of all, $T > 1$, $S < 0$ and $1 - T > -S$ (*T-driven* game), then $\rho = \frac{1-T}{S} > 1$.

Secondly, notice that:

$$Q_v = \frac{(\rho-1)k_v\overline{x}_v^* + k_v}{\rho} > 0,$$

since $k_v \geq 1$, $\overline{x}_v^* \in [0,1]$ and $\rho > 1$. Therefore, β_v is positive when it belongs to the set $(Q_v, \rho Q_v)$.

Since $\beta_v < \rho Q_v$, then $\rho Q_v - \beta_v > 0$. Dividing the last inequality by $(\rho-1)\beta_v$, we get:

$$\frac{\rho Q_v - \beta_v}{(\rho-1)\beta_v} > 0. \qquad (7)$$

Similarly, since $\beta_v > Q_v$, then $\rho Q_v < \rho\beta_v$. Moreover, subtracting β_v from both left and right side of the inequality, we get $\rho Q_v - \beta_v < \rho\beta_v - \beta_v$, or equivalently, $\rho Q_v - \beta_v < (\rho-1)\beta_v$. Dividing the last inequality by $(\rho-1)\beta_v$, we get:

$$\frac{\rho Q_v - \beta_v}{(\rho-1)\beta_v} < 1. \qquad (8)$$

The proof is concluded by observing that:

$$
\begin{aligned}
x_v^* &= \frac{k_v}{\beta_v}\overline{x}_v^* - \frac{S}{1-T-S}\left(1-\frac{k_v}{\beta_v}\right) \\
&= \frac{k_v}{\beta_v}\overline{x}_v^* - \left(\frac{1-T-S}{S}\right)^{-1}\left(1-\frac{k_v}{\beta_v}\right) \\
&= \frac{k_v}{\beta_v}\overline{x}_v^* - \left(\frac{1-T}{S}-1\right)^{-1}\left(1-\frac{k_v}{\beta_v}\right) \\
&= \frac{k_v}{\beta_v}\overline{x}_v^* - (\rho-1)^{-1}\left(1-\frac{k_v}{\beta_v}\right) \\
&= \frac{k_v}{\beta_v}\overline{x}_v^* - \frac{1}{\rho-1}\left(1-\frac{k_v}{\beta_v}\right) \\
&= \frac{k_v}{\beta_v}\overline{x}_v^* - \frac{1}{\rho-1}\left(\frac{\beta_v-k_v}{\beta_v}\right) \\
&= \frac{(\rho-1)k_v\overline{x}_v^* + k_v - \beta_v}{(\rho-1)\beta_v} \\
&= \frac{\rho Q_v - \beta_v}{(\rho-1)\beta_v}. \qquad (9)
\end{aligned}
$$

Therefore, inequalities (7) and (8) imply that:

$$x_v^* \in (0,1).$$

□

Theorem 2. *Consider an S-driven game. Let* $\rho = \frac{S}{1-T}$ *and let* $Q_v = (1-\rho)k_v\overline{x}_v^* + \rho k_v$. *Then*

$$\beta_v \in \left(\frac{Q_v}{\rho}, Q_v\right) \iff x_v^* \in (0,1).$$

Proof. First of all, $T > 1$, $S < 0$ and $1-T < -S$ (*S-driven* game), then $\rho = \frac{S}{1-T} > 1$.

Secondly, notice that:

$$Q_v = (1-\rho)k_v\overline{x}_v^* + \rho k_v = k_v\left((1-\rho)\overline{x}_v^* + \rho\right) = k_v\left(\overline{x}_v^* + \rho(1-\overline{x}_v^*)\right) > 0,$$

since $k_v \geq 1$, $\overline{x}_v^* \in [0,1]$ and $\rho > 1$. Therefore, β_v is positive when it belongs to the set $\left(\frac{Q_v}{\rho}, Q_v\right)$.

Since $\beta_v > \frac{Q_v}{\rho}$, then $Q_v - \rho\beta_v < 0$. Dividing the last inequality by $(1-\rho)\beta_v$, we get:

$$\frac{Q_v - \rho\beta_v}{(1-\rho)\beta_v} > 0, \qquad (10)$$

where the last inequality holds since $(1-\rho)\beta_v < 0$.

Similarly, since $\beta_v < Q_v$, then $\beta_v - \rho\beta_v < Q_v - \rho\beta_v$, or equivalently, $(1-\rho)\beta_v < Q_v - \rho\beta_v$. Dividing the last inequality by $(1-\rho)\beta_v$, we get:

$$\frac{Q_v - \rho\beta_v}{(1-\rho)\beta_v} < 1. \qquad (11)$$

The proof is concluded by observing that:

$$
\begin{aligned}
x_v^* &= \frac{k_v}{\beta_v}\overline{x}_v^* - \frac{S}{1-T-S}\left(1-\frac{k_v}{\beta_v}\right) \\
&= \frac{k_v}{\beta_v}\overline{x}_v^* - \left(\frac{1-T-S}{S}\right)^{-1}\left(1-\frac{k_v}{\beta_v}\right) \\
&= \frac{k_v}{\beta_v}\overline{x}_v^* - \left(\frac{1-T}{S}-1\right)^{-1}\left(1-\frac{k_v}{\beta_v}\right) \\
&= \frac{k_v}{\beta_v}\overline{x}_v^* - \left(\frac{1}{\rho}-1\right)^{-1}\left(1-\frac{k_v}{\beta_v}\right) \\
&= \frac{k_v}{\beta_v}\overline{x}_v^* - \left(\frac{\rho}{1-\rho}\right)\left(1-\frac{k_v}{\beta_v}\right) \\
&= \frac{k_v}{\beta_v}\overline{x}_v^* - \left(\frac{\rho}{1-\rho}\right)\left(\frac{\beta_v-k_v}{\beta_v}\right) \\
&= \frac{(1-\rho)k_v\overline{x}_v^* + \rho k_v - \rho\beta_v}{(1-\rho)\beta_v} \\
&= \frac{Q_v - \rho\beta_v}{(1-\rho)\beta_v}. \qquad (12)
\end{aligned}
$$

Therefore, inequalities (10) and (11) imply that:

$$x_v^* \in (0,1).$$

□

The stability of a steady state $x_v^* \in (0,1)$ can be studied by observing that the sign of $\dot{x}_v$ in equation (4) depends only on the term $\frac{\partial \phi_v(\mathbf{x})}{\partial x_v} - \beta_v f_v(x_v)$; according to equation (6), this term is linear with respect to x_v, and hence the stability of x_v^* depends on the slope of this straight line. Specifically, the slope is equal to

$$-\beta_v(1-T-S).$$

Since $\beta_v > 0$, then we can have two cases:

- the slope is positive when the game is *T-driven*. In this case, the point x_v^* acts as a repeller;
- the slope is negative when the game is *S-driven*. In this case, the point x_v^* acts as an attractor.

These results are reported in Figure 2 of the main paper.

2 Global emergence of cooperation in the EGN equation with self-regulations

The global emergence of cooperation is reached when all members of a social network turn their strategies to cooperation. Therefore, the asymptotic stability of $\mathbf{x}^*_{ALLC}$, as well as the instability of $\mathbf{x}^*_{ALLD}$, have a fundamental role in this context. In order to study the stability of steady states $\mathbf{x}^*_{ALLC}$ and $\mathbf{x}^*_{ALLD}$, we start by analyzing their linear stability. Moreover, an appropriate Lyapunov function is found, for proving that, under certain hypotheses, $\mathbf{x}^*_{ALLC}$ is also globally asymptotically stable. Finally, different hypotheses are used for identifying a Lyapunov function proving that $\mathbf{x}^*_{ALLD}$ is globally asymptotically stable.

2.1 Asympotic stability of $\mathbf{x}^*_{ALLC}$

Recall that the spectrum of $\mathbf{J}(\mathbf{x}^*)$ characterizes the linear stability of any steady state $\mathbf{x}^*$ [S8].

The following results hold.

Theorem 3. *If $\beta_v > k_v \ \forall v \in \mathcal{V}$, then $\mathbf{x}^*_{ALLC}$ is asymptotically stable.*

Proof. As shown before, the Jacobian matrix evaluated for $\mathbf{x}^*_{ALLC}$ is diagonal. Then, the elements on the diagonal of the Jacobian matrix correspond to its eigenvalues and they are defined as follows:

$$j_{v,v}(\mathbf{x}^*_{ALLC}) = \lambda_v = (T-1)(k_v - \beta_v).$$

Since $\beta_v > k_v \ \forall v \in \mathcal{V}$ and $T > 1$, all the eigenvalues are negative. Thus, $\mathbf{x}^*_{ALLC}$ is asymptotically stable. □

Theorem 4. *If $\exists v \in \mathcal{V} : \beta_v > k_v$, then $\mathbf{x}^*_{ALLD}$ is unstable.*

Proof. The eigenvalues of the Jacobian matrix relative to the steady state $\mathbf{x}^*_{ALLD}$ are:

$$j_{v,v}(\mathbf{x}^*_{ALLD}) = \lambda_v = S(k_v - \beta_v).$$

If the hypothesis of the theorem are fulfilled, since $S < 0$, then there is at least one positive eigenvalue, implying that $\mathbf{x}^*_{ALLD}$ is an unstable steady state. □

These results are summarized as follows: defection dominates over cooperation. Then, if the system does not present any internal feedback mechanism (i.e. $\beta_v = 0 \ \forall v \in \mathcal{V}$), the whole social network will converge to $\mathbf{x}^*{}_{ALLD}$ (cooperation vanishes). Anyway, using $\beta_v > k_v$ for all the members of the population, $\mathbf{x}^*{}_{ALLD}$ is destabilized and $\mathbf{x}^*{}_{ALLC}$ becomes attractive.

2.2 Global asymptotic stability of $\mathbf{x}^*_{ALLC}$

Theorems 3 and 4 prove that under suitable condition, $\mathbf{x}^*_{ALLC}$ is asymptotically stable and $\mathbf{x}^*_{ALLD}$ is unstable. Anyway, this is not sufficient to prove the global emergence of cooperation. Indeed, there can be some other steady states in Θ which may be also attractive. Nevertheless, a Lyapunov function [S9] for the steady state $\mathbf{x}^*_{ALLC}$ on the set $\mathbf{x} \in (0,1]^N$ can be found.

Adapting the approach presented in [S10, S11] to the SR-EGN equation, we consider the following function:

$$V(\mathbf{x}) = -\sum_{v=1}^{N} \log(x_v),$$

for $\mathbf{x} \in (0,1]^N$. Notice that $V(\mathbf{x}^*_{ALLC}) = 0$, and $V(\mathbf{x}) > 0 \ \forall \mathbf{x} \neq \mathbf{x}^*_{ALLC}$.

Moreover, the time derivative of $V(\mathbf{x})$ is defined as follows:

$$\begin{aligned} \dot{V}(\mathbf{x}) = \frac{\partial V(\mathbf{x})}{\partial t} &= \sum_{v=1}^{N} \frac{\partial V(\mathbf{x})}{\partial x_v} \dot{x}_v = \\ &= -\sum_{v=1}^{N} \frac{1}{x_v} x_v (1 - x_v) \left(\frac{\partial \phi_v(\mathbf{x})}{\partial x_v} - \beta_v f_v(x_v) \right) = \\ &= \sum_{v=1}^{N} (x_v - 1) \left(\frac{\partial \phi_v(\mathbf{x})}{\partial x_v} - \beta_v f_v(x_v) \right). \end{aligned} \tag{13}$$

Clearly, $\dot{V}(\mathbf{x}^*_{ALLC}) = 0$.

Starting from these premises, if $\dot{V}(\mathbf{x}) < 0$ for all $\mathbf{x} \in (0,1]^N \setminus \{\mathbf{x}^*_{ALLC}\}$, then $V(\mathbf{x})$ is a Lyapunov function.

Let's introduce the following quantities:

$$\psi = \inf_{y \in (0,1]} \left[(1-T-S)y + S\right], \tag{14}$$

$$\xi = \sup_{y \in (0,1]} \left[(1-T-S)y + S\right], \tag{15}$$

and

$$\rho = \frac{\psi}{\xi}.$$

It is easy to show that:

$$\psi = \min\{1-T, S\} = -\max\{T-1, -S\},$$

and

$$\xi = \max\{1-T, S\} = -\min\{T-1, -S\},$$

and hence:

$$\rho = \frac{\max\{T-1, -S\}}{\min\{T-1, -S\}}.$$

Interestingly, the parameter ρ is equal to the ratio between the maximum and minimum of two "driving forces", namely the temptation to defect $(T-1)$ and the disadvantage of being defected $(-S)$. Therefore, we can distinguish two cases:

- for a *T-driven* game, since $T-1 > -S$, then $\psi = 1-T$, $\xi = S$ and $\rho = \dfrac{1-T}{S}$;
- for a *S-driven* game, since $T-1 < -S$, then $\psi = S$, $\xi = 1-T$ and $\rho = \dfrac{S}{1-T}$.

The following result holds.

Theorem 5. *If $\beta_v > \rho k_v$ $\forall v \in \mathcal{V}$, then $V(\mathbf{x})$ is a Lyapunov function.*

Proof. It is straightforward to observe that:

$$\frac{\partial \phi_v(\mathbf{x})}{\partial x_v} = \sum_{w=1}^{N} a_{v,w}[(1-T-S)x_w + S] \geq \sum_{w=1}^{N} a_{v,w}\psi = k_v\psi. \tag{16}$$

Similarly, since $\beta_v > 0$, we get that:

$$\beta_v f_v(x_v) = \beta_v[(1-T-S)x_v + S] \leq \beta_v\xi. \tag{17}$$

Joining (16) and (17) together, we get that:

$$\frac{\partial \phi_v(\mathbf{x})}{\partial x_v} - \beta_v f_v(x_v) \geq k_v\psi - \beta_v\xi.$$

Moreover, notice that:

$$\beta_v > \rho k_v \Rightarrow \beta_v > \frac{\psi}{\xi}k_v \Rightarrow k_v\psi - \beta_v\xi > 0,$$

and hence

$$\frac{\partial \phi_v(\mathbf{x})}{\partial x_v} - \beta_v f_v(x_v) > 0 \ \forall v \in \mathcal{V}. \tag{18}$$

According to equations (13) and (18), since $x_v - 1 < 0$ for all $\mathbf{x} \in (0,1]^N \setminus \{\mathbf{x}^*_{ALLC}\}$, we guarantee that $\dot{V}(\mathbf{x}) < 0$ for all $\mathbf{x} \in (0,1]^N \setminus \{\mathbf{x}^*_{ALLC}\}$. Hence, $V(\mathbf{x})$ is a Lyapunov function. □

Corollary 1. *If* $\beta_v > \rho k_v$*, then*

$$\lim_{t\to\infty} x_v(t) = 1$$

.

Proof. This is a direct consequence of inequality (18). □

2.3 Global asymptotic stability of $\mathbf{x}^*_{ALLD}$

Consider the following function for the steady state $\mathbf{x}^*_{ALLD}$:

$$V(\mathbf{x}) = -\sum_{v=1}^{N} \log(1 - x_v),$$

for $\mathbf{x} \in [0, 1)^N$. Notice that $V(\mathbf{x}^*_{ALLD}) = 0$, and $V(\mathbf{x}) > 0\ \forall \mathbf{x} \neq \mathbf{x}^*_{ALLD}$.

The time derivative of $V(\mathbf{x})$ is defined as follows:

$$\begin{aligned}
\dot{V}(\mathbf{x}) = \frac{\partial V(\mathbf{x})}{\partial t} &= \sum_{v=1}^{N} \frac{\partial V(\mathbf{x})}{\partial x_v}\dot{x}_v = \\
&= \sum_{v=1}^{N} \frac{1}{1 - x_v} x_v(1 - x_v)\left(\frac{\partial \phi_v(\mathbf{x})}{\partial x_v} - \beta_v f_v(x_v)\right) = \\
&= \sum_{v=1}^{N} x_v \left(\frac{\partial \phi_v(\mathbf{x})}{\partial x_v} - \beta_v f_v(x_v)\right). \qquad (19)
\end{aligned}$$

Clearly, $\dot{V}(\mathbf{x}^*_{ALLD}) = 0$.

Starting from these premises, if $\dot{V}(\mathbf{x}) < 0$ for all $\mathbf{x} \in [0, 1)^N \setminus \{\mathbf{x}^*_{ALLD}\}$, then $V(\mathbf{x})$ is a Lyapunov function.

The following result holds.

Theorem 6. *If* $\beta_v < \frac{1}{\rho} k_v\ \forall v \in \mathcal{V}$*, then* $V(\mathbf{x})$ *is a Lyapunov function.*

Proof. It is straightforward to see that:

$$\frac{\partial \phi_v(\mathbf{x})}{\partial x_v} = \sum_{w=1}^{N} a_{v,w}[(1 - T - S)x_w + S] \leq \sum_{w=1}^{N} a_{v,w}\xi = k_v\xi. \qquad (20)$$

Similarly, since $\beta_v > 0$, we get that:

$$\beta_v f_v(x_v) = \beta_v[(1 - T - S)x_v + S] \geq \beta_v\psi. \qquad (21)$$

Joining (20) and (21) together, we get that:

$$\frac{\partial \phi_v(\mathbf{x})}{\partial x_v} - \beta_v f_v(x_v) \leq k_v\xi - \beta_v\psi.$$

Moreover, notice that:

$$\beta_v < \frac{1}{\rho} k_v \Rightarrow \beta_v < \frac{\xi}{\psi} k_v \Rightarrow k_v\xi - \beta_v\psi < 0,$$

and hence

$$\frac{\partial \phi_v(\mathbf{x})}{\partial x_v} - \beta_v f_v(x_v) < 0\ \forall v \in \mathcal{V}. \qquad (22)$$

According to equations (19) and (22), since $x_v > 0$ for all $\mathbf{x} \in [0, 1)^N \setminus \{\mathbf{x}^*_{ALLD}\}$, we guarantee that $\dot{V}(\mathbf{x}) < 0$ for all $\mathbf{x} \in [0, 1)^N \setminus \{\mathbf{x}^*_{ALLD}\}$. Hence, $V(\mathbf{x})$ is a Lyapunov function. □

Corollary 2. *If* $\beta_v < \frac{1}{\rho} k_v$, *then*

$$\lim_{t\to\infty} x_v(t) = 0$$

.

Proof. This is a direct consequence of inequality (22). □

2.4 Game transitions

The previous results highlight the effectiveness of the self-regulating term in promoting cooperation. In this Section we show that this fact is due to transitions between different games, occurring when the system parameters are changed. We start from the generic formulation of the replicator equation for the two-strategy case, as proposed in [S10]:

$$\dot{x} = x(1-x)\left[(\sigma_C + \sigma_D)x - \sigma_D\right], \tag{23}$$

where σ_C and σ_D are the elements of the diagonal payoff matrix

$$\mathbf{B} = \begin{bmatrix} \sigma_C & 0 \\ 0 & \sigma_D \end{bmatrix}.$$

The sign of the parameters σ_C and σ_D rules the dynamics of equation (23) as follows:

- $\sigma_C < 0$ and $\sigma_D > 0$: Prisoner's dilemma game (PD), for which defection is the only dominant strategy;
- $\sigma_C > 0$ and $\sigma_D > 0$: Stag Hunt game (SH), where cooperation is the best response to cooperation, and defection is the best response to defection;
- $\sigma_C < 0$ and $\sigma_D < 0$: Chicken game (CH), where cooperation is the best response to defection, and vice versa;
- $\sigma_C > 0$ and $\sigma_D < 0$: Harmony game (HA), for which cooperation is the only dominant strategy.

It is worthwhile to notice that $\sigma_C < 0$ models the temptation to defect, while $\sigma_D > 0$ models the fear to be betrayed.

We can rewrite the SR-EGN equation according to the structure of equation (23):

$$\begin{aligned} \dot{x}_v &= x_v(1-x_v)\{k_v\left[(1-T-S)\bar{x}_v + S\right] - \beta_v\left[(1-T-S)x_v + S\right]\} \\ &= x_v(1-x_v)\left[(\sigma_C^v + \sigma_D^v)x_v - \sigma_D^v\right]. \end{aligned}$$

Then, we have the following relationships:

$$\begin{cases} \sigma_C^v + \sigma_D^v = -\beta_v(1-T-S) \\ \sigma_D^v = -\left\{k_v[(1-T-S)\overline{x}_v + S] - \beta_v S\right\} \end{cases} \Rightarrow$$

$$\Rightarrow \begin{cases} \sigma_C^v = k_v\left[(1-T-S)\overline{x}_v + S\right] - \beta_v(1-T) \\ \sigma_D^v = -k_v\left[(1-T-S)\overline{x}_v + S\right] + \beta_v S \end{cases}. \tag{24}$$

σ_C^v and σ_D^v characterize the equivalent game of player v. They depend on game parameters T and S, degree k_v and self-regulating factor β_v. Interestingly, they depend also on the strategy of the equivalent player $\overline{x}_v$. This fact implies that the values of σ_C^v and σ_D^v change not only by changing the system's parameters, but also over time, according to the dynamics of $\overline{x}_v$, thus producing dynamical game transitions. Hereafter, we report the conditions for which these transitions occur.

2.4.1 *T-driven* case: σ_C^v

We establish the conditions for which the sign of σ_C^v is independent or dependent on the on the level of cooperation of his neighborhood $\overline{x}_v$. According to equation (24), we have

$$\sigma_C^v = k_v\left[(1-T-S)\overline{x}_v + S\right] - \beta_v(1-T).$$

We distinguish three cases.

1. $\sigma_C^v > 0$ for any $\overline{x}_v \in [0,1]$ if $k_v\left[(1-T-S)\overline{x}_v + S\right] > \beta_v(1-T)$. Using equation (14), since in this case $\psi = 1-T$, we have that:

$$k_v\left[(1-T-S)\overline{x}_v + S\right] \geq k_v\psi = k_v(1-T)\ \forall \overline{x}_v \in [0,1].$$

Then

$$\sigma_C^v \geq k_v(1-T) - \beta_v(1-T) = (1-T)(k_v - \beta_v)\ \forall \overline{x}_v \in [0,1].$$

Since $T > 1$, we get the following result:

$$\beta_v > k_v \Rightarrow \sigma_C^v > 0\ \forall \overline{x}_v \in [0,1].$$

2. $\sigma_C^v < 0$ if $k_v\left[(1-T-S)\overline{x}_v + S\right] < \beta_v(1-T)$ for any $\overline{x}_v \in [0,1]$. Using equation (15), since in this case $\xi = S$, we have that:

$$k_v\left[(1-T-S)\overline{x}_v + S\right] \leq k_v\xi = k_v S\ \forall \overline{x}_v \in [0,1].$$

Since $\rho = \frac{1-T}{S}$, then:

$$\sigma_C^v \leq k_v S - \beta_v(1-T) = S(k_v - \beta_v\rho)\ \forall \overline{x}_v \in [0,1].$$

By hypothesis, $S < 0$, and hence we get the following result:

$$\beta_v < \frac{k_v}{\rho} \Rightarrow \sigma_C^v < 0\ \forall \overline{x}_v \in [0,1].$$

3. The sign of σ_C^v depends on $\overline{x}_v$ when $\beta_v \in \left[\frac{k_v}{\rho}, k_v\right]$.

Noticing that

$$\sigma_C^v > 0 \Leftrightarrow k_v((1-T-S)\overline{x}_v + S) - \beta_v(1-T) > 0,$$

and dividing by $S < 0$, since $\rho = \frac{1-T}{S}$, we get:

$$k_v((\rho-1)\overline{x}_v + 1) - \beta_v\rho < 0 \Rightarrow \overline{x}_v < \frac{\beta_v\rho - k_v}{k_v(\rho-1)}.$$

Summarizing:

- $\sigma_C^v > 0$ for $\overline{x}_v < \dfrac{\beta_v\rho - k_v}{k_v(\rho-1)}$;
- $\sigma_C^v < 0$ for $\overline{x}_v > \dfrac{\beta_v\rho - k_v}{k_v(\rho-1)}$.

Additionally, by the definition of σ_C^v we have that

$$\frac{\partial \sigma_C^v}{\partial \overline{x}_v} = k_v(1-T-S) < 0,$$

then for the *T-driven* case σ_C^v decreases as $\overline{x}_v$ increases.

2.4.2 *T-driven* case: σ_D^v

We establish the conditions for which the sign of σ_D^v is independent or dependent on the on the level of cooperation of his neighborhood $\overline{x}_v$. According to equation (24), we have

$$\sigma_D^v = -k_v\left[(1-T-S)\overline{x}_v + S\right] + \beta_v S.$$

We distinguish three cases.

1. $\sigma_D^v > 0$ for any $\overline{x}_v \in [0,1]$ if $k_v\left[(1-T-S)\overline{x}_v + S\right] < \beta_v S$.
   Using equation (15), since $\xi = S$, we have that

   $$k_v\left[(1-T-S)\overline{x}_v + S\right] \leq k_v\xi = k_v S \ \forall \overline{x}_v \in [0,1].$$

   Then,

   $$\sigma_D^v \geq -k_v S + \beta_v S = S(\beta_v - k_v) \ \forall \overline{x}_v \in [0,1].$$

   Since $S < 0$, we get the following result:

   $$\beta_v < k_v \Rightarrow \sigma_D^v > 0 \ \forall \overline{x}_v \in [0,1].$$

2. $\sigma_D^v < 0$ for any $\overline{x}_v \in [0,1]$ if $k_v\left[(1-T-S)\overline{x}_v + S\right] > \beta_v S$. Using equation (14), since $\psi = 1-T$ we have that:

   $$k_v\left[(1-T-S)\overline{x}_v + S\right] \geq k_v\psi = k_v(1-T) \ \forall \overline{x}_v \in [0,1],$$

   Then, since $\rho = \frac{1-T}{S}$, we have:

   $$\sigma_D^v \leq -k_v(1-T) + \beta_v S = -S(k_v\rho - \beta_v) \ \forall \overline{x}_v \in [0,1].$$

   Since, moreover, $S < 0$, we get the following result:

   $$\beta_v > k_v\rho \Rightarrow \sigma_D^v < 0 \ \forall \overline{x}_v \in [0,1].$$

3. For $\beta_v \in [k_v, k_v\rho]$ the sign of σ_D^v depends on $\overline{x}_v$. Starting from

   $$\sigma_D^v > 0 \Leftrightarrow k_v((1-T-S)\overline{x}_v + S) - \beta_v S < 0,$$

   and dividing the inequality by $S < 0$, since $\rho = \frac{1-T}{S}$, we get:

   $$k_v((\rho-1)\overline{x}_v + 1) - \beta_v > 0 \Rightarrow \overline{x}_v > \frac{\beta_v - k_v}{k_v(\rho-1)}.$$

   Summarizing:

   - $\sigma_D^v > 0$ for $\overline{x}_v > \dfrac{\beta_v - k_v}{k_v(\rho-1)}$;
   - $\sigma_D^v < 0$ for $\overline{x}_v < \dfrac{\beta_v - k_v}{k_v(\rho-1)}$.

   Additionally, by the definition of σ_D^v we have that:

   $$\frac{\partial\sigma_D^v}{\partial\overline{x}_v} = -k_v(1-T-S) > 0$$

   for the *T-driven* case, and hence σ_D^v increases as $\overline{x}_v$ increases.

Table 1 reports a summary of the results on game transitions for the *T-driven* case.

Condition	σ_C^v	σ_D^v	Set of v	Equivalent game
$0 < \beta_v < \dfrac{k_v}{\rho}$	< 0	> 0	$\mathcal{D}$	PD
$\dfrac{k_v}{\rho} < \beta_v < k_v$	$\in \mathbb{R}$	> 0	$\mathcal{U}$	PD or SH
$k_v < \beta_v < k_v\rho$	> 0	$\in \mathbb{R}$	$\mathcal{U}$	SH or HA
$\beta_v > k_v\rho$	> 0	< 0	$\mathcal{C}$	HA

Table 1: Game transitions in the *T-driven* case.

2.4.3 *S-driven* case: σ_C^v

We establish the conditions for which the sign of σ_C^v is independent or dependent on the on the level of cooperation of his neighborhood $\overline{x}_v$. According to equation (24), we have

$$\sigma_C^v = k_v\left[(1-T-S)\overline{x}_v + S\right] - \beta_v(1-T).$$

We distinguish three cases.

1. $\sigma_C^v > 0$ for any $\overline{x}_v \in [0,1]$ if $k_v\left[(1-T-S)\overline{x}_v + S\right] > \beta_v(1-T)$. Using equation (14), since $\psi = S$, we have

$$k_v\left[(1-T-S)\overline{x}_v + S\right] \geq k_v\psi = k_v S \ \forall \overline{x}_v \in [0,1].$$

   Therefore, since $\rho = \frac{S}{1-T}$, we have

$$\sigma_C^v \geq k_v S - \beta_v(1-T) = (1-T)(k_v\rho - \beta_v) \ \forall \overline{x}_v \in [0,1].$$

   Moreover, since $T > 1$, we get the following result:

$$\beta_v > k_v\rho \Rightarrow \sigma_C^v > 0 \ \forall \overline{x}_v \in [0,1].$$

2. $\sigma_C^v < 0$ for any $\overline{x}_v \in [0,1]$ if $k_v\left[(1-T-S)\overline{x}_v + S\right] < \beta_v(1-T)$. Using equation (15), since $\xi = 1-T$, we have

$$k_v\left[(1-T-S)\overline{x}_v + S\right] \leq k_v\xi = k_v(1-T) \ \forall \overline{x}_v \in [0,1].$$

   Then:

$$\sigma_C^v \leq k_v(1-T) - \beta_v(1-T) = (1-T)(k_v - \beta_v) \ \forall \overline{x}_v \in [0,1].$$

   By hypothesis, $T > 1$, and hence we get the following result:

$$\beta_v < k_v \Rightarrow \sigma_C^v < 0 \ \forall \overline{x}_v \in [0,1].$$

3. For $\beta_v \in [k_v, k_v\rho]$, the sign of σ_C^v depends on $\overline{x}_v$. Starting from

$$\sigma_C^v > 0 \Leftrightarrow k_v((1-T-S)\overline{x}_v + S) - \beta_v(1-T) > 0,$$

and dividing the inequality by $1-T<0$, since $\rho=\frac{S}{1-T}$, we get:

$$k_v((1-\rho)\overline{x}_v+\rho)-\beta_v<0 \Rightarrow \overline{x}_v>\frac{k_v\rho-\beta_v}{k_v(\rho-1)}.$$

Summarizing:

- $\sigma_C^v>0$ for $\overline{x}_v>\dfrac{k_v\rho-\beta_v}{k_v(\rho-1)}$;
- $\sigma_C^v<0$ for $\overline{x}_v<\dfrac{k_v\rho-\beta_v}{k_v(\rho-1)}$.

Moreover, since:

$$\frac{\partial\sigma_C^v}{\partial\overline{x}_v}=k_v(1-T-S)>0$$

for the *S-driven* case, then σ_C^v increases as $\overline{x}_v$ increases.

2.4.4 *S-driven* case: σ_D^v

We establish the conditions for which the sign of σ_D^v is independent or dependent on the level of cooperation of his neighborhood $\overline{x}_v$. According to equation (24), we have

$$\sigma_D^v=-k_v\left[(1-T-S)\overline{x}_v+S\right]+\beta_v S.$$

We distinguish three cases.

1. $\sigma_D^v>0$ for any $\overline{x}_v\in[0,1]$ if $k_v\left[(1-T-S)\overline{x}_v+S\right]<\beta_v S$. Using equation (15), since $\xi=1-T$, we have

   $$k_v\left[(1-T-S)\overline{x}_v+S\right]\leq k_v\xi=k_v(1-T)\ \forall\overline{x}_v\in[0,1].$$

   Therefore, recalling that $\rho=\frac{S}{1-T}$, we get:

   $$\sigma_D^v\geq -k_v(1-T)+\beta_v S=(1-T)(\beta_v\rho-k_v)\ \forall\overline{x}_v\in[0,1].$$

   Since $T>1$, we get the following result:

   $$\beta_v<\frac{k_v}{\rho}\Rightarrow\sigma_D^v>0\ \forall\overline{x}_v\in[0,1].$$

2. $\sigma_D^v<0$ for any $\overline{x}_v\in[0,1]$ if $k_v\left[(1-T-S)\overline{x}_v+S\right]>\beta_v S$. Using equation (14), since $\psi=S$, we have that:

   $$k_v\left[(1-T-S)\overline{x}_v+S\right]\geq k_v\psi=k_v S\ \forall\overline{x}_v\in[0,1].$$

   Then:

   $$\sigma_D^v\leq -k_v S+\beta_v S=-S(k_v-\beta_v)\ \forall\overline{x}_v\in[0,1].$$

   Since $S<0$, we get the following result:

   $$\beta_v>k_v\Rightarrow\sigma_D^v<0\ \forall\overline{x}_v\in[0,1].$$

Condition	σ_C^v	σ_D^v	Set of v	Game
$0 < \beta_v < \dfrac{k_v}{\rho}$	< 0	> 0	$\mathcal{D}$	PD
$\dfrac{k_v}{\rho} < \beta_v < k_v$	< 0	$\in \mathbb{R}$	$\mathcal{U}$	PD or CH
$k_v < \beta_v < k_v\rho$	$\in \mathbb{R}$	< 0	$\mathcal{U}$	CH or HA
$\beta_v > k_v\rho$	> 0	< 0	$\mathcal{C}$	HA

Table 2: Game transitions in the *S-driven* case.

3. For $\beta_v \in \left[\frac{k_v}{\rho}, k_v\right]$, the sign of σ_D^v depends on $\overline{x}_v$. Starting from

$$\sigma_D^v > 0 \Leftrightarrow k_v((1 - T - S)\overline{x}_v + S) - \beta_v S < 0,$$

and dividing the inequality by $1 - T < 0$, since $\rho = \frac{S}{1-T}$, we get:

$$k_v((1 - \rho)\overline{x}_v + \rho) - \beta_v\rho > 0 \Rightarrow \overline{x}_v < \frac{\rho(k_v - \beta_v)}{k_v(\rho - 1)}.$$

Summarizing:

- $\sigma_D^v > 0$ for $\overline{x}_v < \dfrac{\rho(k_v - \beta_v)}{k_v(\rho - 1)}$;
- $\sigma_D^v < 0$ for $\overline{x}_v > \dfrac{\rho(k_v - \beta_v)}{k_v(\rho - 1)}$.

Additionally, by the definition of σ_D^v we have that:

$$\frac{\partial \sigma_D^v}{\partial \overline{x}_v} = -k_v(1 - T - S) < 0$$

for the *T-driven* case, and hence σ_D^v decreases as $\overline{x}_v$ increases.

Table 2 reports a summary of the results on game transitions for the *S-driven* case.

References

[S1] Santos, F.C., Pacheco, J.M. & Lenaerts, T. Evolutionary dynamics of social dilemmas in structured heterogeneous populations. *P. Natl. Acad. Sci. USA* **103**, 3490–3494 (2006).

[S2] Killingback, T., Doebeli, M. & Knowlton, N. Variable investment, the continuous prisoner's dilemma, and the origin of cooperation. *Proceedings of the Royal Society of London B: Biological Sciences* **266**, 1723-1728 (1999).

[S3] Doebeli, M., Hauert, C. & Killingback, T. The evolutionary origin of cooperators and defectors. *Science* **306**, 859-862 (2004).

[S4] Zhong, W., Kokubo, S. & Tanimoto, J. How is the equilibrium of continuous strategy game different from that of discrete strategy game? *BioSystems* **107**, 88-94 (2012).

[S5] Madeo, D. & Mocenni, C. Game Interactions and dynamics on networked populations. *IEEE T. Automat. Contr.* **60**, 1801-1810 (2015).

[S6] G. Iacobelli, D. Madeo and C. Mocenni. Lumping evolutionary game dynamics on networks. *Journal of Theoretical Biology*, vol. 407, pp. 328-338 (2016).

[S7] Schuster, P., Sigmund, K., Hofbauer, J., Gottlieb, R. & Merz, P. Selfregulation of behaviour in animal societies. *Biol. Cybern.* **40**, 17-25 (1981).

[S8] Strogatz, S.H. *Nonlinear Dynamics and Chaos: With Applications to Physics, Biology, Chemistry and Engineering* (Westview Press, Boulder, CO, Usa, 2001).

[S9] Khalil, H. K. *Nonliner systems* (NJ: Prentice-Hall, 2002).

[S10] Weibull, J. *Evolutionary Game Theory* (Cambridge, MA: MIT Press, 1995).

[S11] Hofbauer, J. & Sigmund, K. *Evolutionary Games and Population Dynamics* (Cambridge, UK: Cambridge Univ. Press, 1998).
